# In Methuselah's Mould

**DOI:** 10.1371/journal.pbio.0020012

**Published:** 2004-01-20

**Authors:** Bill O'Neill

## Abstract

Is aging inevitable or can it be "cured"? Recent work from many different fields of science is now providing clues into why we age and how long we might live

The pathologist makes do with red wine until an effective drug is available, the biochemist discards the bread from her sandwiches, and the mathematician indulges in designer chocolate with a clear conscience. The demographer sticks to vitamin supplements, and while the evolutionary biologist calculates the compensations of celibacy, the population biologist transplants gonads, but so far only those of his laboratory mice. Their common cause is to control and extend the healthy lifespan of humans. They want to cure ageing and the diseases that come with it.

“I would take resveratrol if it were feasible,” notes David Sinclair, assistant professor of pathology at Harvard Medical School in Boston, Massachusetts. In the meantime, he adds, “I do enjoy a glass of red wine about once a day.” It was Sinclair's laboratory, in association with a commercial partner, that revealed last August how the team had identified for the first time a group of simple organic molecules capable of extending lifespan. The most proficient of the group is resveratrol, the plant polyphenol found in red wine, and its discovery as a potential elixir to combat ageing represents another extraordinary advance in a decade of discoveries that have revolutionised the field.

*“These molecules will be useful for treating diseases associated with ageing, like diabetes and Alzheimer's.”*

## Extending Life

Although the life-enhancing effects of Sinclair's polyphenols are so far confined to the baker's yeast Saccharomyces cerevisiae, the work suggests that researchers are only one small step from making a giant leap for humankind. “People imagined that it might have been possible, but few people thought that it was going to be possible so quickly to find such things,” says Sinclair. The field of ageing research is buzzing.

**Figure 1:**
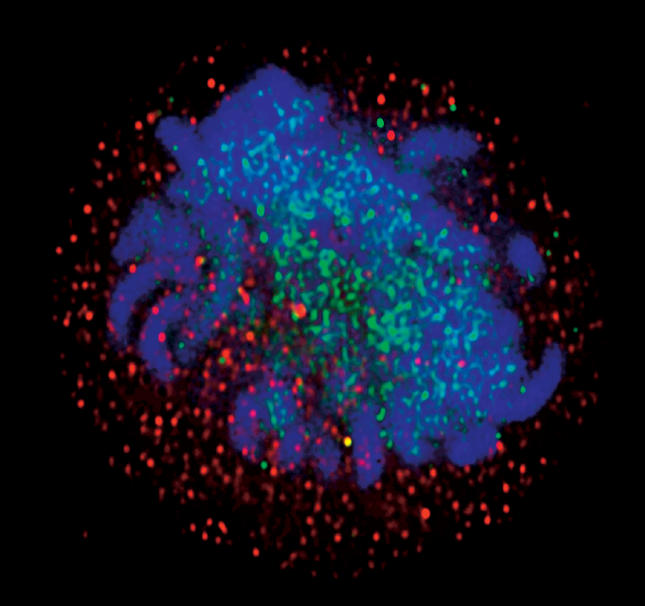
SIRT-1 Deacetylase—the Human Enzyme That Promotes Cell Survival—in a Dividing Human Cell The enzyme is marked in red, and the image is superimposed on acetylated proteins (green) and condensed chromosomes (blue). (Image courtesy of David Sinclair.)

Resveratrol stimulated a known activator of increased longevity in yeast, the enzyme Sir-2, and thereby extended the organism's lifespan by 70% (Box 1). Sir-2 belongs to a family of proteins with members in higher organisms, including SIR-2.1, an enzyme that regulates lifespan in worms, and SIRT-1, the human enzyme that promotes cell survival (Figure 1). Though researchers still do not know whether SIRT-1, or “Sir-2 in humans,” as Sinclair puts it, has anything to do with longevity, there is a good chance that it does, judging by its pedigree. In any event, resveratrol proved to be a potent activator of the human enzyme. This might not be altogether surprising, at least not now, given that the polyphenol is already associated with health benefits in humans, notably the mitigation of such age-related defects as neurodegeneration, carcinogenesis, and atherosclerosis.

“The study came out from a pretty big gamble,” recalls Sinclair, who used the human enzyme to screen and identify molecules that he expected would also stimulate those related enzymes in lower organisms. Unlike SIRT-1, these related enzymes are known to increase longevity when activated, usually by restricting the organism's calorie intake. Not only did they find “a whole collection of related polyphenols that activate ‘Sir-2 from humans,’ … but we put them onto yeast, justbeing the simplest model, and amazingly [they] did what we were hoping [they] would do,” recalls Sinclair. “But it was a real long shot.”

Now there's great eagerness in the Sinclair laboratory to complete and publish related research, notably by replicating the yeast work in higher organisms. “We have very promising results in Drosophila, which is a huge jump from a yeast cell,” says Sinclair. “So we're very encouraged by that.” Publication of these results is imminent. The team has also quickly broadened its horizons and is already testing the polyphenols on mouse disease models. “We think we may have tapped into a cell survival and defence programme [and] that these molecules will be useful for treating diseases associated with ageing, like diabetes and Alzheimer's,” says Sinclair. He hopes to publish the diabetes results by mid-2004 and those for Alzheimer's by the end of the year. Harvard and BIOMOL Research Laboratories, its commercial partner based in Pennsylvania, have already filed a patent application for the use of “synthetic related molecules” to combat diseases of ageing—an application, Sinclair adds, “very much linked to the [polyphenols] paper.”

There's been a radical shift in attitude towards ageing, says Sinclair. Before the 1990s, “people thought that we were a lot like cars, that we would just rust and breakdown—nothing we could do about it. The new idea is that there are pathways that can boost our defences against ageing—the ‘ageing-can-be-regulated’ discovery … that genes can control ageing [and] that there are pathways that [we can use to] slow down the process,” he says. “If that's true—and it really seems to be true for a lot of organisms—if it's true for us, it really means that there is hope that we will be able, one day, to find small molecules that can alter these pathways.”

## How Long Could We Live?

Sinclair expects to see such developments within his lifetime, but he ridicules the notion that humans will experience anything like the 70% extension to lifespan of his cultured yeast. “It'll be great if we can just give people an extra five years and have less disease in their old age and make it less painful,” he says. “We won't be seeing any Methuselahs around,” he insists.

On his side are James Vaupel, one of Europe's leading demographers, and Marc Mangel, a mathematical modeller at the University of California at Santa Cruz. “Since 1840, life expectancy has been going up at 2.5 years per decade and will continue at this rate, maybe a little faster,” says Vaupel, head of the Laboratory of Survival and Longevity at the Max Planck Institute for Demographic Research in Rostock, Germany. Women in Japan currently have the highest average life expectancy of 85, he notes: “So the figure could be 100 in six decades, but not 500.” There's remarkably little people can do even if they want to live as long as possible, he says. “Give up smoking, lose weight, don't drive when drunk, install a smoke detector, take regular exercise,” suggests Vaupel, who insists he does them all, as well as taking vitamin supplements.

*“You look at these worms and think, ‘Oh my God, these worms should be dead.’ But they're not. They're moving around.”*

Mangel sees the problem of assessing the limitations of ageing research as fairly straightforward. Mathematical models, he says, could solve it by linking demographic properties and physiological developments. “We've had a separation of the biology of ageing and the demography of ageing, and they need to come together again,” notes Mangel, whose personal anti-ageing regime involves taking “a dose of anti-oxidant chocolate with a good feeling.”

But Cythnia Kenyon, whose laboratory reported in October that it had generated a 6-fold increase in the lifespan of its nematodes, is not so sure about the limitations. “You look at these worms and think, ‘Oh my God, these worms should be dead.’ But they're not. They're moving around…. Once you get your brain wrapped around that … then you start thinking, oh my goodness, so lifespan is something you can change—it's plastic. Then who knows what the limit is?” (Cynthia Kenyon has recorded video clips of the superstars of her lab, Caenorhabditis elegans, to show how long-lived mutant nematodes are as vigorous as normal young adults [Videos 1–4].)

Warming to the theme, Kenyon hypothesises: “If you'd asked me many generations ago, when we were actually common precursors of worms and flies, ‘Cynthia, you have a two-week lifespan, do you think that you could [live longer]?’ And if I'd told you, ‘Well, I think our descendants will live 1,000 times longer,’ you'd have said, ‘Oh, come on!’ But we do. It happened,” she notes.

“Who knows what you could do in people?” Kenyon muses. “I don't want to go on record saying that it's not possible in people because I don't see why it wouldn't be…. I'm certainly not imagining that my company in the next few years is going to come up with a compound that can make people live to be 500. That seems just preposterous.” So the timescale is millions of years? “No, not necessarily,” she insists, “because once we understand the mechanism, then we can intervene and see what we can accomplish.”

**Box 1. Model Systems for Ageing**

Yeast, as a model system for ageing, is at a distinct disadvantage. It lacks an endocrine system, and yet much research indicates that the key to longevity is control of hormones such as insulin and insulin-like growth factor 1 (IGF-1), as well as their downstream pathways and associated tissues, including the reproductive network.

But David Sinclair, whose laboratory used yeast to show how an elixir might extend life, remains sanguine. “If it doesn't have an endocrine system, we can't understand cell-to-cell communication, but not all of ageing is just communication,” he says. “There are things that occur inside the cells that provide longevity, and that's where yeast can be applied.”

Sinclair, assistant professor of pathology at Harvard Medical School, discovered a group of polyphenols that cause the human enzyme SIRT-1 and its homologues in lower organisms, including Sir-2 in yeast, to deacetylate the p53 protein and its homologues, notably the histones H3 and H4, in yeast.

“Our findings are that the activation of the pathway downregulates p53's ability to cause cell death,” he notes. Although p53, the tumour suppressor, is known to be involved in programmed cell death, it is not known whether SIRT-1 has any role in ageing. So Sinclair “went straight back to yeast to prove the principle of longevity extension.”

He found that deacetylisation of histones in yeast caused the DNA that's wrapped around them to become more compact and thus more stable. “DNA stability is key to longevity, and Sir-2 promotes that,” says Sinclair. “We don't know yet whether it's the same in humans.”

Cynthia Kenyon, meanwhile, sees worms as the optimal model for helping to substantiate the links to humans. Besides the nematodes' being multicellular organisms with endocrine systems, she also notes that their short lifespan of around 20 days is a big advantage: “You can do lots of experiments with them.” Mice, which have short lifespans for mammals, still live two years, and long-lived mice for three or four years, she notes. And the advantage of fruit flies? “It's good to use more than one animal.”

Kenyon, professor of biochemistry and biophysics at the University of California at San Francisco, has focused on decoding the role of genes in ageing, notably *daf-2*, whose receptor is similar to those for insulin and IGF-1 in humans and inhibits ageing, and *daf-16*, which promotes it.

“The DAF-2 receptor activates a highly conserved PI-3-kinase, the PDK/Akt pathway, and that pathway affects ageing, at least in part, by inhibiting the activity of the DAF-16 transcription factor,” says Kenyon. “It does so by phosphorylating DAF-16 and inhibiting its entry into the nucleus.” She adds: “We think that the DAF-2 pathway has another way of influencing ageing … but we don't know what this other way is.”

In the long-lived mutants, which are defective in the *daf-2* receptor gene or in the genes encoding downstream signalling components, such as the PI-3-kinase, DAF-16's activity in the nucleus leads to the changes in expression of a wide variety of downstream genes, between 100 and 200, estimates Kenyon. Her studies show, she says, that a large number of those genes influence ageing.

## Signalling Life and Sweet 16

Kenyon, professor of biochemistry and biophysics at the University of California at San Francisco, is among the key contributors responsible for showing that a single gene, and subsequently many genes, can change an organism's lifespan.

“It is inconceivable … that a life-extending therapy will ever be developed that is able to extend life independent of every other change.”

In a seminal paper published a decade ago, Kenyon's laboratory showed that mutations in the *daf-2* gene doubled the lifespan of the nematode C. elegans. *daf-2* encodes a receptor that is similar to those for insulin and insulin-like growth factor-1 (IGF-1) in humans; this hormone receptor normally speeds up ageing in worms, but the mutations inhibit its action and enable the organisms to live longer. Before the results appeared, there was a “very negative attitude” towards ageing research, recalls Kenyon. Since then, and especially over the past few years in response to later findings, graduate students have been scrambling for a chance to work in her laboratory. “You can't believe the difference—there was such resistance to it,” she says. “*daf-2* made a huge difference.” But then so did her subsequent research in the field.

Among her most significant findings is the identification of many more longevity genes; the results, published in July, derive directly from her early work on *daf-2*. “We discovered that in order for long-lived worms to live so long, they need another gene called *daf-16*,” recalls Kenyon. “*daf-16* is kind of the opposite of *daf-2*, in the sense that it promotes longevity and youthfulness … so we call it ‘sweet 16.’” *daf-16* encodes a transcription factor that controls the expression of more than 100 genes. “They don't do just one thing, they do many things,” says Kenyon. They can act as anti-oxidants (to prevent damage from oxygen radicals), as chaperones (to prevent misfolded proteins from forming aggregates), as antimicrobials (to protect against bacteria and fungi), and as metabolic agents.

“So the picture that emerges is that the way the insulin/IGF-1 hormone system produces these enormous effects on lifespan is by coordinating the expression of many genes that do different things to affect lifespan, each of which on its own has only a small effect,” notes Kenyon. “It's as though *daf-2* and *daf-16*, the regulators, would be the conductors of an orchestra. They bring together the flutes and the violins and the French horns, each of which do different things, and they make them all work together in concert.”

Kenyon is unequivocal about the bottom line: “Now we have a whole set of genes whose biochemical functions we can be working on to understand more about the actual mechanisms of ageing.” Complementary results in flies and mammals persuade her to be more explicit. “The common ancestor of worms, flies, and mice must have had an insulin/IGF-1-like hormone system that controlled ageing. And that ability has been maintained. So the question is, has [that ability] been lost in humans? I think it's quite likely that it will also function in humans, but there isn't a direct demonstration yet that that's the case.”

Nevertheless, the discoveries about the role of the insulin/IGF-1 pathway in ageing have had a profound impact on her own lifestyle, which includes a tendency to discard the bread from sandwiches and eat only the toppings of pizzas (Box 2). “I'm on a low-carb diet. I gave my worms glucose, and it shortened their lifespan. [The diet] makes sense because it keeps your insulin levels down,” she says.

“Caloric restriction extends lifespan of mice, and so does the insulin/IGF-1 pathway,” she notes. Indeed, starting a low-calorie diet at any point in adulthood appears to help fruit flies live longer, according to research in Britain published last September. “What we don't know for sure in mice,” Kenyon continues, “is whether the two pathways are different or the same.”

While much ageing research focuses on these two influences, she says that there are another two areas of investigation. Her laboratory reported in December 2002 that inhibiting the respiration of mitochondria in developing worms increased longevity, but that it had no effect in adult worms, for reasons still unexplained, she says. Further microarray analysis is underway to pinpoint whether the cause simply lies downstream of the insulin/IGF-1 pathway or whether it is something different altogether.

## The Price of Life

Then there's research looking at the effects on lifespan of changes to an organism's reproductive system. For Kenyon, such work often involves a battle to convince sceptics that longevity is not a trade-off with fertility. Four years ago, her laboratory reported that killing germ cells increases the lifespan of worms by 60%, but only because, she stresses, it affects endocrine signalling and not because it prevents reproduction. Further research, published last year, showed quite clearly, she says, that ageing and reproduction are controlled independently of one another. And as for her recent work on infertile worms, which lived six times as long as normal following the removal of their entire reproductive systems, she says: “If we could intervene in the hormone signalling pathways directly, we think the animals would still live six times as long as normal, but would be fertile as well.”

Jim Carey is one of those “trade-off” sceptics. He is a population biologist at the University of California at Davis and his research, on the effect on life expectancy of replacing the ovaries of old mice with ovaries from younger mice, is intended to complement Kenyon's work. But he insists that “an honest discussion of lifespan extension must include consideration of tradeoffs.” Many manipulations that extend lifespan in model systems, whether genetic or dietary, for example, ignore or gloss over the side effects, such as permanent sterility, huge weight loss, distorted organ-to-body ratios, or major behavioural aberrations, he notes. “It is inconceivable to me that a life-extending therapy will ever be developed that is able to extend life independent of every other change,” he concludes. “All life systems are interlinked and hierarchically integrated at all levels, so to talk about life extension using analogies with a car warranty concept is wrong-headed.”

Another “trade-off” sceptic takes a different tack. As Armand Leroi puts it: “During occasional periods of involuntary celibacy I have thought, well, I may not be getting laid, but at least I shall live to a miserable and solitary old age.” Leroi, an evolutionary biologist at Imperial College of Science, Technology, and Medicine in London, offers an optimistic appraisal of the chances of finding a cure for ageing in his new book about the effects of genetic variety on the human body. He sees ageing simply as a collection of curable diseases: “There is no obvious impediment to that advance, nothing to make us think that human beings have a fixed lifespan.”

**Box 2. Practise What You Preach**

Cynthia Kenyon's eating habits are defined by her ageing research on worms. “There's a lot of these diets … and what they all have in common is *low carb*—actually, low glycaemic index carbs,” she says. “That's not eating the kind of carbohydrates where the sugar gets into your bloodstream very quickly [and stimulates production of insulin].”

No desserts. No sweets. No potatoes. No rice. No bread. No pasta. “When I say ‘no,’ I mean ‘no, or not much,’” she notes. “Instead, eat green vegetables. Eat the fruits that aren't the sweet fruits, like melon.” Bananas? “Bananas are a little sweet.” Meat? “Meat, yes, of course. Avocados. All vegetables. Nuts. Fish. Chicken. That's what I eat. Cheese. Eggs. And one glass of red wine a day.”

Kenyon, professor of biochemistry and biophysics at the University of California at San Francisco, has been on her diet for two-and-a-half years. “I did it because we fed our worms glucose and it shortened their lifespan.”

But the diet is unproven, she cautions, and she's not recommending it for all. Nevertheless, she's pleased with its performance for her. “I have a fabulous blood profile. My triglyceride level is only 30, and anything below 200 is good.”

Kenyon is angered by the general lack of nutritional knowledge: “It's a little bit embarrassing to say that scientists actually don't know what you should eat…. We can target particular oncogenes, but we don't know what you should eat. Crazy,” she says.

Does her dieting represent a return to scientists experimenting on themselves? “I don't think so—you have to eat something, and you just have to make your best judgement. And that's my best judgement. Plus, I feel better. Plus, I'm thin—I weigh what I weighed when I was in college. I feel great —you feel like you're a kid again. It's amazing.”

**Video 1:** Normal Nematodes at Day 1 of Adulthood (Video used by permission from Cynthia Kenyon.)

**Video 2:** Long-Lived *daf-2* Mutants at Day 1 of Adulthood (Video used by permission from Cynthia Kenyon.)

**Video 3:** Normal Nematodes at Day 13 of Adulthood The worm on the left is dead. (Video used by permission from Cynthia Kenyon.)

**Video 4:** A Long-Lived *daf-2* Mutant at Day 13 of Adulthood (Video used by permission from Cynthia Kenyon.)

## Abbreviation

IGF-1: insulin-like growth factor-1
